# *TFG::ALK* fusion in ALK positive large B-cell lymphoma: a case report and review of literature

**DOI:** 10.3389/fonc.2023.1174606

**Published:** 2023-05-25

**Authors:** Andrew Xiao, Nahid Shahmarvand, Alexandra Nagy, Jyoti Kumar, Jessica Van Ziffle, Patrick Devine, Franklin Huang, Lhara Lezama, Peng Li, Robert S. Ohgami

**Affiliations:** ^1^ Department of Pathology, University of California San Francisco, San Francisco, CA, United States; ^2^ Syneos Health, Morrisville, NC, United States; ^3^ Department of Pathology, Stanford University, Stanford, CA, United States; ^4^ Department of Pathology and Laboratory Medicine, Memorial Sloan Kettering Cancer Center, New York, NY, United States; ^5^ Department of Pathology, Kaiser Permanente, Los Angeles, CA, United States; ^6^ Department of Pathology, University of Utah and ARUP Laboratories, Salt Lake City, UT, United States

**Keywords:** ALK+ large B-cell lymphoma, TFG, ALK, ALK translocation, ALK fusion

## Abstract

Anaplastic lymphoma kinase (ALK) positive large B-cell lymphoma (ALK+ LBCL) is an aggressive and rare subtype of B-cell lymphoma. Patients typically present with advanced clinical stage disease and do not respond to conventional chemotherapy; the median overall survival is 1.8 years. The genetic landscape of this entity remains poorly understood. Here we report a unique case of ALK+ LBCL harbouring a rare *TFG::ALK* fusion. Targeted next-generation sequencing showed no significant single nucleotide variants, insertions/deletions, or other structural variants beyond the *TFG::ALK* fusion; deep deletions of *FOXO1*, *PRKCA*, and the *MYB* locus were also detected. Our case report draws attention to this rare disease, highlights a need for larger genetic profiling studies, and focuses on the pathogenesis and potential therapeutic targets of this aggressive disease. To our knowledge, this is the first report of a *TFG::ALK* fusion in ALK+ LBCL.

## Introduction

Anaplastic lymphoma kinase (ALK) positive large B cell lymphoma (ALK+ LBCL) is a rare aggressive subtype of large B-cell lymphoma (LBCL), accounting for less than 1% of all diffuse large B-cell lymphoma (DLBCL) (1–3). It was first reported in 1997 by Delsol and colleagues (4) but has since been described in only small or individual case reports; to this date fewer than 200 cases have been reported in the literature. This disease occurs in both adult and pediatric patients with the median age at diagnosis of 38 years [range of 9-90 years (5)] but most often is seen in immunocompetent male patients with a male:female ratio of 4.2 (5). Advanced disease with cervical lymph node and/or extranodal involvement is a common presentation of this disease. Patients often do not respond to treatment with CHOP (cyclophosphamide, doxorubicin, vincristine, and prednisolone), and median survival is 1.8 years (5).

The diagnosis of ALK+ LBCL is challenging because of its rarity and its morphologic and immunophenotypic resemblance to other hematopoietic and non-hematopoietic neoplasms (6). Histologically, it can show sinusoidal or a diffuse growth pattern consisting of sheets of large monomorphic immunoblastic or plasmablastic cells. By immunophenotype, the tumor cells importantly express ALK protein, and commonly co-express plasma cell markers such as CD38, CD138, VS38c, multiple myeloma oncogene 1 (MUM1); are negative for T-cell markers, and most cases are negative or only partially express B-cell markers (CD19, CD20, CD79a, PAX5) (1, 7, 8).

ALK overexpression in ALK+ LBCL is due to its translocation which generates a fusion protein. The most common translocation is t(2;17)(p23;q23) involving clathrin (*CLTC*) on chromosome 17q23 and *ALK* on chromosome 2p23 resulting in a *CLTC::ALK* fusion translocation (1, 9). ALK fusion proteins lead to ligand independent dimerization of the intracellular tyrosine kinase domain of ALK (10) and result in constitutive activation which promotes oncogenesis (11). Here we report the first case of an ALK+ LBCL with *TFG::ALK* fusion and describe the unique clinical, morphologic, immunophenotypic, and molecular features.

## Materials and methods

This study was approved by the University of California, San Francisco (UCSF) institutional review board. Clinical information was obtained from Kaiser Permanente, Los Angeles. All hematoxylin and eosin (H&E) and immunohistochemistry slides were reviewed for this study. Immunohistochemistry was performed on whole slide sections to confirm the diagnosis of ALK+ LBCL, including ALK-1, Oct2, P53, c-MYC, BOB1, CD45RB (LCA), CD79a, CD10, Bcl-6, CD138, MUM-1, IgG, INI-1. Ki-67, Pancytokeratin, CK7, CK20, CK5/6, EMA, P63, P40, CK903, TTF-1, PAX5, CD20, CD30, CD2, CD3, CD4, CD5, CD7, CD8, CD43, CD21, CD23, Bcl-2, Cyclin D1, CD56, CD57, CD1a, CD68 (PGM1), SOX10, Melan-A, S100, HMB-45, SALL4, synaptophysin, chromogranin, INSM1, TdT, myeloperoxidase, CD34, CD31, ERG, desmin, myogenin, SF-1, GFAP, HHV-8, EBV by *in situ* hybdridization (ISH), IgM, IgA, IgD, and Kappa and Lambda ISH.

Capture-based next-generation sequencing (NGS) was performed at the UCSF Clinical Cancer Genomics Laboratory with UCSF500, a targeted sequencing panel consisting of all coding regions of 529 cancer-related genes and selected introns from 47 genes. Analysis of single nucleotide variants, insertion/deletions, structural variants including gene fusions, genome-wide copy number, and zygosity analysis, with a total sequencing footprint of 2.8 Mb was performed and bioinformatic analysis was performed using custom pipelines as previously described (12, 13).

## Case report

A 23-year-old previously healthy male developed 7 months of nasal congestion and obstruction. At the time of presentation, he admitted to fatigue, diaphoresis, abdominal pain, and nausea. A physical exam revealed right cervical lymphadenopathy. A PET scan demonstrated a 2.3 cm left nasopharyngeal mass, bilateral cervical lymphadenopathy, and right retropharyngeal lymphadenopathy. A subsequent brain MRI scan was negative and endoscopy demonstrated a friable nasopharyngeal mass which was biopsied.

**Figure 1:**
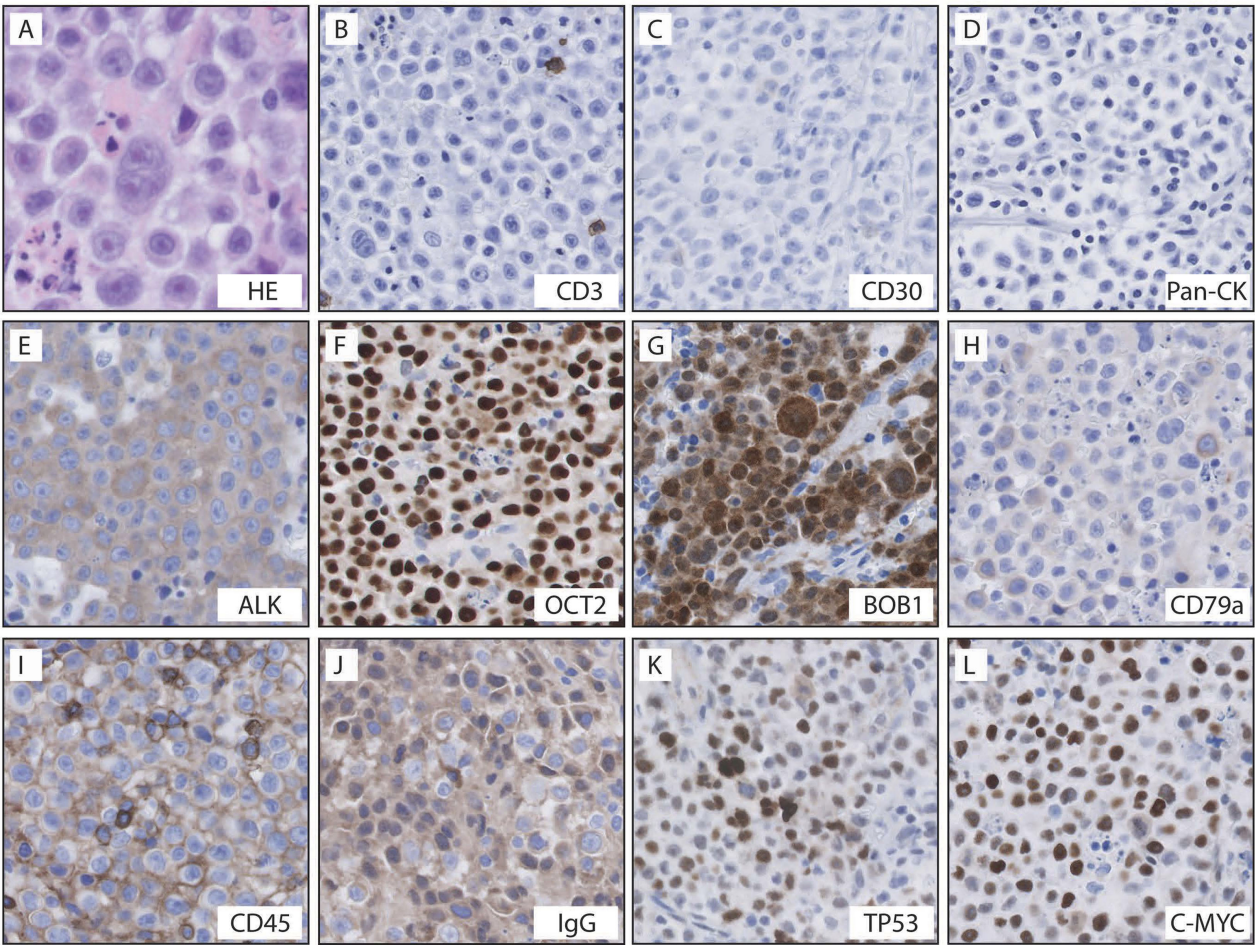
Histologic features and immunohistochemical stains in this case of ALK+ LBCL. Histology demonstrated sheets of large monomorphic immunoblastic or plasmablastic cells with moderate to abundant amounts of lightly eosinophilic cytoplasm and abundant necrosis **(A)**. The tumor cells were negative for CD3 **(B)**, CD30 **(C)**, and pancytokeratin **(D)**; the tumor cells were positive for ALK **(E)**, Oct2 **(F)**, BOB1 **(G)**, dim CD79a **(H)**, CD45RB **(I)**, IgG **(J)**, TP53 **(K)**, and c-MYC **(L)**.

H&E stained slides of the nasopharyngeal mass showed sheets of large cells with round to oval nuclei, dispersed chromatin and prominent, often single, centrally located nucleoli (**Figure 1A**). These cells had moderate to abundant amounts of lightly eosinophilic cytoplasm. Mitotic figures were easily identified and there was abundant necrosis.

A broad panel of immunostains was performed and the neoplastic cells were positive for ALK, Oct2, c-MYC, BOB1, CD45RB (LCA), CD79a (dim), CD10 (dim), Bcl-6 (subset), CD138, MUM-1, IgG. (**Figures 1B–L**). TP53 was positive in 30-40% of tumor cells. INI-1 staining was intact. Kappa and Lambda ISH show kappa light chain restriction on cells. Ki-67 demonstrated 70-80% proliferation in the tumor cells. The following immunohistochemical stains were negative in the neoplastic cells: pancytokeratin, CK7, CK20, CK5/6, EMA, P63, P40, CK903, TTF-1, PAX5, CD20, CD30, CD3, CD4, CD5, CD7, CD8, CD43, CD21, CD23, Bcl-2, Cyclin D1, CD56, CD57, CD1a, CD68 (PGM1), SOX10, Melan-A, S100, HMB-45, SALL4, synaptophysin, chromogranin, INSM1, TdT, myeloperoxidase, CD34, CD31, ERG, desmin, myogenin, SF-1, GFAP, HHV-8, EBV-ISH, IgM, IgA, IgD. A staging bone marrow biopsy showed no evidence of involvement by LBCL.

## Molecular results

**Figure 2:**
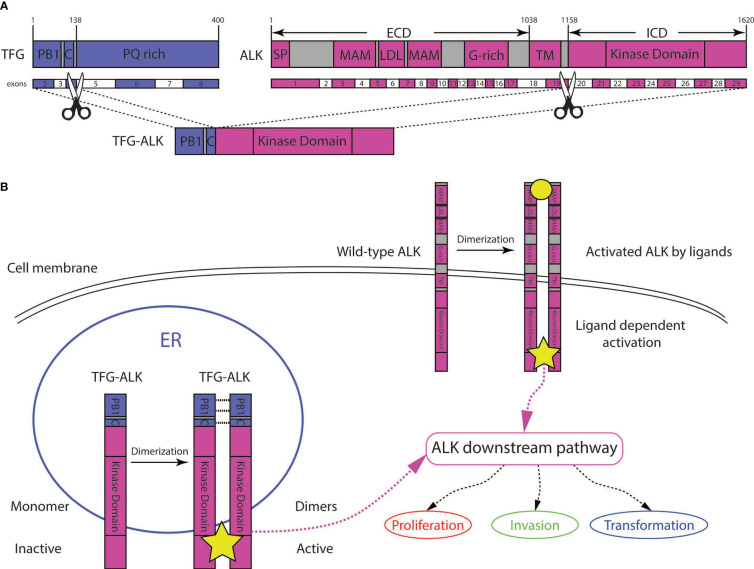
Next-generation sequencing using a DNA targeted sequencing panel identified a *TFG::ALK1* fusion involving exons 1-4 in *TFG* and exons 20-29 in *ALK* **(A)**. The TFG-ALK fusion protein results in downstream up-regulation of the ALK pathway implicated in many oncogenic cellular pathways, including cell survival, transformation, invasion and proliferation **(B)**. PB1, Phox and Bem1p domain; C, coiled coil region; PQ rich, proline and glutamine rich region; SP, signal peptide, MAM, Meprin, A5 protein and protein tyrosine phosphatase Mu domain; LBD, low density lipoprotein receptor domain; G-rich, glycrine-rich region, TM, transmembrane domain; Kinase domain, tyrosine kinase domain; ER, endoplasmic reticulum.

NGS using a DNA targeted sequencing panel identified a *TFG::ALK1* fusion involving exons 1-4 of *TFG* and exons 20-29 of *ALK* (**Figure 2A**). No Tier 1 single nucleotide variants (SNV) were identified. One *PRDM1* p.N421S variant of uncertain significance (VUS) was identified at a variant allele frequency (VAF) of 48%, likely representing a germline variant given the VAF. Deep deletions at *FOXO1*, *MYB* and *PRKCA* were identified. A custom microbial analysis pipeline did not identify any microbial organisms.

## Treatment and follow-up

The patient was treated with EPOCH (etoposide phosphate, prednisone, oncovin/vincristine sulfate, cyclophophamide, hydroxydaunomycin/doxorubicin hydrocholoride) and provided pembrolizumab (Keytruda) for maintenance after testing positive for PD-L1. Six months after diagnosis, the patient was refractory to treatment with no significant change upon PET scan.

## Discussion

We report the clinicopathologic features of a rare case of ALK+ LBCL with a *TFG::ALK* fusion analyzed by NGS. *TFG::ALK* fusions have been reported in a case of lung adenocarcinoma, inflammatory myofibroblastic tumor, anaplastic large cell lymphomas, a histiocytic neoplasm, a plasma cell neoplasm, and papillary thyroid carcinomas (**Table 1**) (14–18, 21–26). To our knowledge, this is the first report of a *TFG::ALK* fusion in an ALK+ LBCL.

**Table 1:** Summary of *TFG::ALK* rearranged neoplasms.

Case	Age	Sex	Dx	Site	Size	Stage	FC	IHC
Pt 1	27	M	Lung Adeno	Lung	2 cm	IIB	N/A	ALK+
Pt 2	14	M	IMT	Pelvis	8cm	N/A	N/A	ALK+
Pt 3	49	M	PC	Nasopharyngeal	5.4 cm	N/A		ALK-, CD138+, Kappa+, LCA+, CD117+
Pt 4	19	M	ALCL (common)	LN, SNS	N/A	N/A	N/A	ALK+, CD30+
Pt 5	10	F	ALCL (null)	LN, SNS	N/A	N/A	N/A	ALK+ (cyto), CD30+, CD3+, CD5+, EMA+
Pt 6	21	M	ALK+ HN	Liver, skin, colorectum	N/A	N/A	N/A	ALK+, CD68+, CD163+
Pt 7	10	F	aIMT	Abdominal cavity	N/A	N/A	N/A	ALK+ (cyto)
Pt 8	N/A	N/A	FVPTC	N/A	N/A	N/A	N/A	ALK+ (cyto)

Case	Cytogenetics		Molecular	Treatment	Survival		Remission	Citation
Pt 1	*TFG::ALK* (e1-4);(e20-29)		N/A	N/A	N/A		N/A	(14)
Pt 2	*TFG::ALK* (e1-6); (e20-29)		N/A	N/A	N/A		N/A	(15)
Pt 3	*TFG::ALK* (e1-4); (e20-29)		TP53 p.H178Tfs*69 (VAF 45%), TNFAIP3 p.C27Ffs*44 (VAF 25%), MYO6 p.E953* (VAF 37.6%), HGF p.G674D (VAF 37.6%), GRID1 p.S556Y (VAF 27.5%)	Alectinib	2 years		Yes	(16)
Pt 4	*TFG::ALK* (e1-4); (e20-29)		N/A	N/A	N/A		N/A	(17, 18)
Pt 5	*TFG::ALK* (e1-4); (e20-29)		N/A	German NHL-BFM’90 (19, 20)	16 months		No	(21)
Pt 6	*TFG::ALK* (e1-4); (e20-29)		N/A	Corticosteroids	2 months		N/A	(22)
Pt 7	*TFG::ALK* (e1-5); (e20-29)		N/A	N/A	81 months		N/A	(23)
Pt 8	*TFG::ALK* (e1-5); (e20-29)		N/A	N/A	N/A		N/A	(24)

ALK, anaplastic lymphoma kinase; NHL, non-Hodgkin lymphoma; LN, SNS, lymph node, site not specified; cyto, cytoplasmic; Pt, patient; FC, flow cytometry; IHC, immunohistochemistry; ALCL, anaplastic large cell lymphoma; e, exon; M, male; F, female; N/A, not applicable; HN, histiocytic neoplasm/histiocytosis; aIMT, atypical inflammatory myofibroblastic tumor; Adeno, adenocarcinoma; IMT, inflammatory myofibroblastic tumor; PC, plasmacytoma

Trk-fused gene (*TFG*) was originally identified as the fusion partner of the *NRTK1* gene (27). Subsequent studies identified *TFG* as a partner to other genes such as *ALK* (17). Since its early discovery, functional studies have shown that TFG, in octamer form, plays an important role in protein trafficking from the endoplasmic reticulum to the Golgi apparatus (28). Multimerization of TFG is possible due to its N-terminal Phox and Bem1p (PB1) and coiled-coil (CC) regions, interaction domains that allow TFG to self-oligomerize (**Figure 2B**).

In the case of the TFG-ALK fusion protein, presumably the fusion of the TFG PB1 and CC domains to the ALK kinase domain allows for the ALK fusion protein to dimerize, and become constitutively activated (**Figure 2B**). This mechanism was illustrated by *in vitro* experiments demonstrating that expression of the TFG-ALK protein resulted in 1) increased proliferation of cells 2) invasiveness of cells, and 3) transformation in NIH3T3 cells (**Figure 2B**) (11).

It is interesting to note that this particular t(2;3)(p23;q21);*ALK::TFG* fusion is represented so broadly in diverse tumor types originating from various cells (i.e. B-cell, T-cell, plasma cell, myofibroblast, histocyte and lung epithelium). Perhaps this translocation is not responsible for cell fate and other somatic genetic abnormalities drive the mutated cancer stem cell towards B-cell, T-cell or other cell type differentiation. Another possibility is that the translocation may occur in a very early pluripotent stem cell which is local to or migrates to different tissues and then the cancer cells differentiate according to anatomic site. Further *in vivo* large-scale studies could help understand the mechanism of disease here.

ALK+ LBCL is a rare neoplasm and most cases show an ALK fusion partner t(2;17)(p23;q23) involving clathrin (*CLTC*), which results in a *CTCL::ALK* rearrangement. Other identified fusion proteins include nucleophosmin (NPM1)-ALK, SEC31A-ALK, SQSTM1-ALK, RANBP2-ALK, EML4-ALK, and GORASP2-ALK (1, 7, 8).

Studies exploring the genomic landscape of ALK+ LBCL are limited and, to our knowledge, no previous NGS study on ALK+ LBCL has been performed. Our sequencing analysis only demonstrated a *TFG::ALK* fusion and no other pathogenic somatic mutations were identified. In contrast, mutational analysis of 20 diffuse large B-cell lymphoma, not otherwise specified (DLBCL, NOS), using this same panel showed a median single nucleotide variant mutational burden of approximately 7.3 (range 0 to 23). The relatively silent cancer genome of this *TFG::ALK* ALK+ LBCL, save the translocation, is consistent with oncogenically defining translocations seen in many neoplasms where that characteristic driver fusion mutation determines the cellular behavior of a tumor, and further mutations are not critical or necessary for the propagation of that tumor. This driver effect is important to consider as it indicates that inhibition of this central fusion gene and associated pathways could potentially reduce tumor growth. Importantly, studies have shown that *ALK* translocated tumors are highly sensitive to targeted ALK inhibitor therapies (10, 16, 29). Presently, crizotinib is the most well studied ALK inhibitor in ALK+ LBCL and it induces a transient improvement in lymphadenopathy and serum LDH levels, though this has been followed by rapid progression and a survival time of less than 6 months (5). Soumerai et al. evaluated the therapeutic potential of higher potency ALK inhibitors alectinib and lorlatinib in patient-derived xenograft models. They hypothesize that crizotinib resistance in ALK+LBCL may be overcome by these higher potency ALK inhibitors *via* upregulation of bypass signalling pathways possibly engaged by the tumor microenvironment (29).

In conclusion, we present the first report of ALK+ LBL harboring a *TFG::ALK* translocation identified by NGS. Our findings provide novel insight into the pathogenesis of this unique disease process. Further large cohort genetic studies are necessary to expand upon our initial molecular profiling, which may also inform therapeutic management.

## Data availability statement

The original contributions presented in the study are included in the article/supplementary material. Further inquiries can be directed to the corresponding author.

## Ethics statement

Written informed consent was obtained from the participant/patient(s) for the publication of this case report.

## Author contributions

AX, PL and RO wrote the manuscript, conceived of the idea, and analyzed data. NS, JK, and AN contributed to writing the manuscript and analyzing data. JZ, PD, LL and FH edited the manuscript and analyzed data. All authors contributed to the article and approved the submitted version.
